# Lactoferrin as a Non-Hormonal Therapeutic Candidate for Endometriosis: Mechanisms and Future Directions

**DOI:** 10.1007/s43032-026-02059-x

**Published:** 2026-03-05

**Authors:** Akiko Nakamura, Yuji Tanaka, Akie Takebayashi, Tsukuru Amano, Shunichiro Tsuji

**Affiliations:** Department of Obstetrics and Gynaecology, Shiga University of Medical Science, 520-2192/Seta Tsukinowa-cho, Otsu, Shiga Japan

**Keywords:** Lactoferrin, Endometriosis, Inflammation, Oxidative stress, Non‑hormonal therapy

## Abstract

The pathogenesis and progression of endometriosis may involve a complex combination of multiple factors, including chronic inflammation and oxidative stress. Hormonal therapy, the current standard for pharmacotherapy in endometriosis, causes various issues, such as restricting pregnancy during treatment and a high risk of recurrence after treatment discontinuation. This review investigates the use of lactoferrin (LF), a natural iron-binding glycoprotein, and outlines its mechanism of action, its potential as a non-hormonal therapeutic strategy, prospects for clinical application, and associated therapeutic issues. LF exerts anti-inflammatory, iron-chelating, antioxidant effects, and antiproliferative effects by suppressing signaling pathways and exhibits antifibrotic, antiangiogenic, and antibacterial properties. These are thought to be important physiological factors in endometriosis progression, for which LF exhibits a promising therapeutic candidate that could theoretically replace or complement hormone therapy. Observational studies have reported variations in serum and peritoneal fluid concentrations of LF and anti-LF antibodies in patients with endometriosis depending on the endometriosis stage, depicting LF as a potential therapeutic target. Furthermore, an in vitro study demonstrated that LF selectively induced cell cycle arrest in endometriotic stromal cells without affecting that of eutopic endometrial cells. Compared with other non-hormonal therapies, LF has an extremely lower risk of teratogenicity and fetal toxicity and could improve reproductive outcomes and perinatal prognosis, indicating its potential for continuous administration throughout various life stages. Further studies are needed to determine optimal administration routes and dosages for clinical applications.

**Graphical Abstract:**

Multiple factors contribute to the pathogenesis and progression of endometriosis, namely, estrogen-responsive growth, inflammation, oxidative stress, signal-dependent cell proliferation, and infection. Lactoferrin targets these pathways through its multifaceted mechanisms of action and offers distinct advantages for patients with endometriosis who wish to preserve their future fertility. Abbreviations: GnRH, gonadotropin-releasing hormone; OC, oral contraceptives

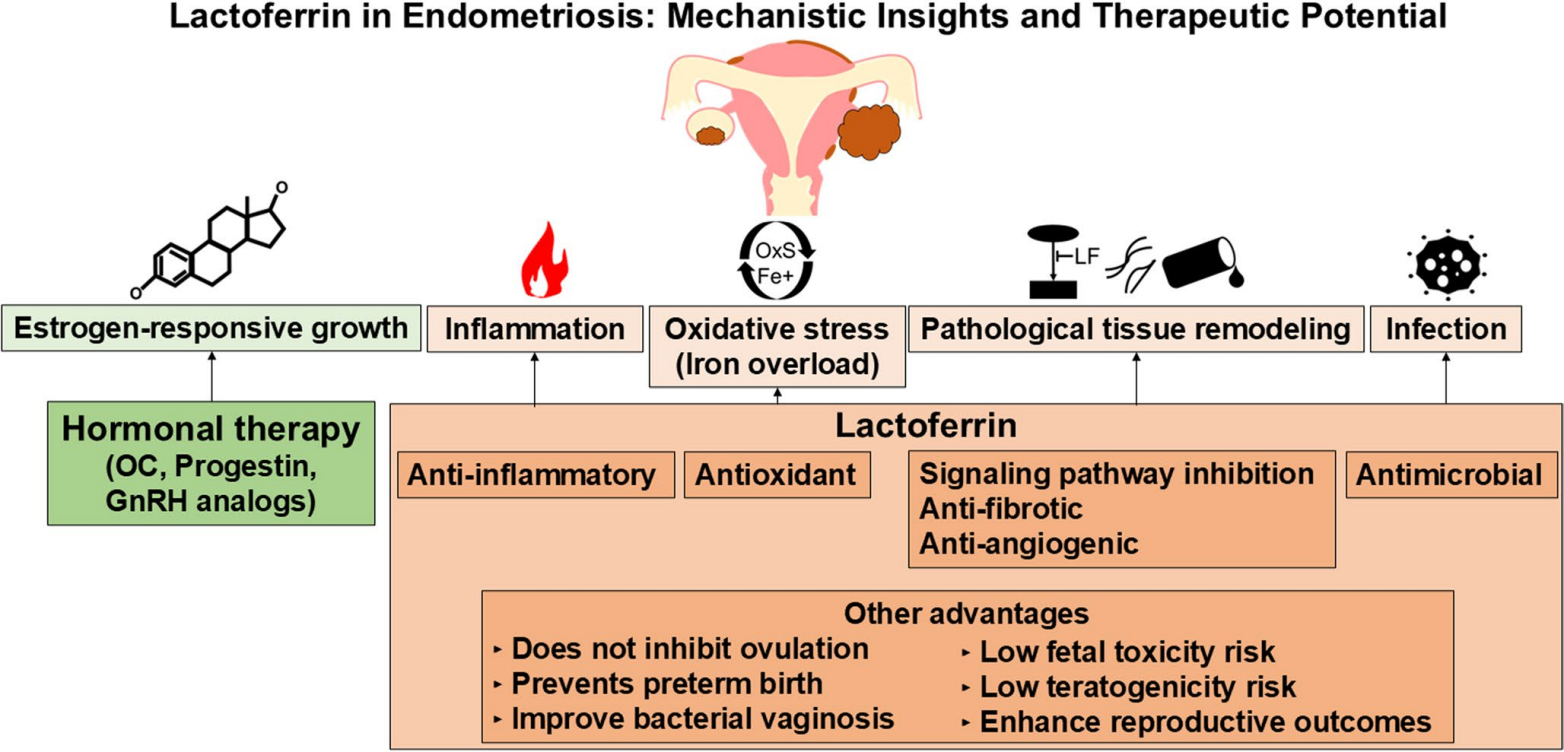

## Introduction

Endometriosis is a chronic, estrogen-dependent inflammatory disease. Its prevalence is high, affecting approximately 5%–10% of women of reproductive age. Its various symptoms include dysmenorrhea, chronic pelvic pain, and even infertility, significantly reducing the quality of life of patients [1]. Various factors are involved in pathogenesis and progression, with immune and inflammatory responses and iron overload-induced oxidative stress being particularly important factors [2–6]. In particular, endometriosis may progress through the activation of signaling pathways (e.g., PI3K/Akt/mTOR, MAPK/ERK, NF-κB, etc.), fibrosis, and angiogenesis, similar to malignant tumors [7–11].

Current pharmacotherapies for endometriosis primarily involve hormone regulation and ovulation suppression, thereby restricting pregnancy but increasing recurrence risk after treatment discontinuation [12]. Resistance to endocrine therapy and associated side effects limit its therapeutic efficacy. Therefore, non-hormonal therapies that can be continuously administered while preserving ovulatory function must be developed.

Against this background, we focus on lactoferrin (LF), a natural iron-binding glycoprotein. LF is abundantly produced by pregnant and postpartum women and occurs in very high concentrations in breast milk [13, 14]. LF has also been rated as GRAS (Generally Recognized as Safe) by U.S. Food and Drug Administration (GRN Nos. 465 and 669) and as a novel food by European Food Safety Authority [15]. It is also recognized as a food ingredient and dietary supplement with established safety. LF has been administered to women planning pregnancy and neonates in various clinical studies [16–19]. LF also exerts anti-inflammatory, antiproliferative, and antioxidant effects. Basic and clinical studies have demonstrated the efficacy of LF in treating chronic and acute inflammatory diseases in the female reproductive tract, including endometritis, cervicitis, and vaginosis [20–23]. Furthermore, LF regulates oxidative stress in the amniotic fluid [24] and can inhibit fibrosis, angiogenesis, and cell proliferation via specific signaling pathways [25–29]. Signaling pathways, such as PI3K/Akt/mTOR, MAPK/ERK, and NF-κB, may be involved in the pathogenesis of endometriosis; LF may be an ideal therapeutic agent.

This review is the first to explore the potential of LF as a non-hormonal therapy for endometriosis. Here, we first outline the epidemiology and pathophysiology of endometriosis and describe the limitations of current hormonal therapies. We then discuss the potential mechanisms of action of LF in endometriosis, focusing on its anti-inflammatory, antiproliferative, and iron-chelating effects on signaling pathways, as well as its inhibition of fibrosis and angiogenesis. Furthermore, we discuss the current challenges and future research prospects for the clinical application of LF (e.g., administration route, pharmacokinetics), thereby contributing to a more comprehensive understanding of non-hormonal therapeutic strategies for endometriosis.

## Methods

**Fig. 1:**
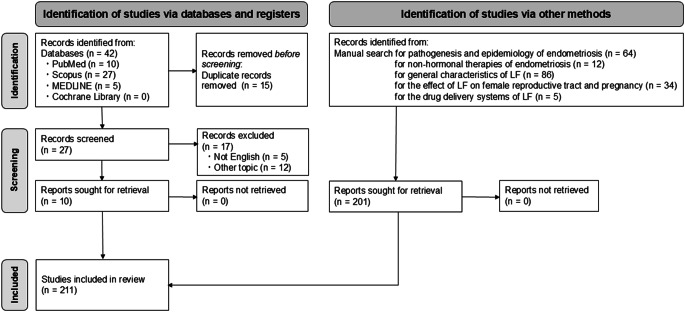
PRISMA flow diagram for the identification, screening, and inclusion of relevant articles

To select articles and set inclusion criteria, a database search was performed in PubMed, Scopus, MEDLINE, and the Cochrane Library for articles published between January 2000 and July 2025 using the keywords “endometriosis” and “lactoferrin.” Two researchers independently conducted the searches. In addition to the database search, we performed a manual search of the reference lists of included articles and key journals. This process aimed to identify articles on conventional hormonal treatments and emerging non-hormonal therapies for endometriosis and to contextualize the potential of LF. We also searched for studies addressing the pathogenesis and epidemiology of endometriosis, the general characteristics of LF, and the effects of LF on the female reproductive tract and pregnancy. Furthermore, to evaluate clinical applicability, we searched for studies on drug delivery systems (DDS) that enhance the bioavailability and therapeutic efficacy of LF. Titles and abstracts were reviewed to assess relevance to the topic. Potentially relevant articles were subjected to full-text review. To meet the inclusion criteria, articles had to be published in peer-reviewed journals written in English and specifically address the involvement of LF in endometriosis and the female reproductive tract, as well as the biological effects of LF. The identified articles and related studies were then synthesized to demonstrate the feasibility of LF therapy for endometriosis (Fig. 1).

### Endometriosis: Epidemiology and Disease Background

Endometriosis is a disease in which endometrial-like tissue grows outside the uterine cavity, such as in the ovaries, pelvic peritoneum, and rectovaginal septum [30, 31]. Approximately 5%–10% of women of reproductive age, particularly between the late 20 s and early 40 s, suffer from this disease [32, 33]. The pathogenesis of endometriosis remains unclear, although some presumed mechanisms include the reflux of menstrual blood and coelomic epithelial metaplasia [12]. Although many women experience menstrual blood reflux, only approximately 10% actually form lesions, suggesting endometriosis development involves a complex combination of genetic predisposition, epigenetic changes, environmental factors, and immunological abnormalities [30, 34–37].

In particular, inflammation contributes significantly to endometriosis development and progression. Neutrophils and macrophages infiltrate into the lesions and continuously secrete proinflammatory cytokines, such as interleukin (IL)−1β, IL-6, and tumor necrosis factor (TNF)-α, as well as various chemokines, forming an inflammatory microenvironment [38, 39]. Increased prostaglandin E2 (PGE2) and cyclooxygenase-2 (COX2) expression has also been observed in preclinical and clinical studies of endometriosis [40–43].

Furthermore, 40%–80% of affected patients also suffer from chronic endometritis, indicating that chronic inflammation may contribute to infertility and worsening clinical symptoms [44–46].

Oxidative stress due to iron overload is also important in the progression of endometriosis. Repeated microbleeds cause excessive iron accumulation in the pelvic cavity, which in turn generates large amounts of reactive oxygen species (ROS) via the Fenton reaction, enhancing inflammation and tissue damage [4–6, 47, 48].

Diseased tissue exhibits increased cell proliferation via various signaling pathways, such as PI3K/Akt/mTOR, MAPK/ERK, and NF-κB [8, 10, 11, 49, 50]. Angiogenesis is promoted by the increased expression of vascular endothelial growth factor (VEGF) and angiopoietin 2 [51]. Activation of TGF-β1/Smad signaling increases the number of α-SMA-positive prefibroblasts (myofibroblasts), promotes collagen deposition and fibrosis, and contributes to adhesion formation [52]. Furthermore, diseased tissue undergoes epithelial–mesenchymal transition (EMT) and exhibits proliferative, invasive, and recurrence properties similar to those of malignant tumors [53, 54].

Some bacteria have been reported to be associated with the pathogenesis and progression of endometriosis, with infection possibly further inducing inflammation and adhesion [55, 56].

Taking all these factors into consideration, endometriosis should be understood as more than just a hormone-dependent disease, but as a complex one that involves chronic inflammation, oxidative stress, abnormal cell proliferation, and infection risk. To address this complex pathology, a multifaceted non-hormonal approach is needed that does not suppress ovulation, controls inflammation and oxidative stress, inhibits cell proliferation, and exerts antibacterial effects.

### Limitations of Current Treatments and Rationale for Non-Hormonal Therapies

The current treatment options for endometriosis are surgery and medication. Conservative surgery for endometriosis is effective in alleviating clinical symptoms; however, this approach presents a high risk of postoperative decline in ovarian function. Furthermore, 20%–50% of patients experience recurrence within 2–5 years postoperatively, necessitating long-term postoperative pharmacotherapy [57–59].

The first-line pharmacotherapy for endometriosis is hormone therapy, which includes oral contraceptives (OC), progestins (e.g., dienogest, levonorgestrel-releasing intrauterine system), and gonadotropin‑releasing hormone (GnRH) analogs (agonists or antagonists). All of these options lower estrogen levels, thereby shrinking lesions and relieving associated pain [60, 61]. GnRH analogs are the most potent estrogen suppressors and therefore exert a high therapeutic effect; however, long-term use poses the risk of osteoporosis and cardiovascular events [62, 63]. Progestin monotherapy is effective against endometriosis mainly by creating a low-estrogen environment while exhibiting anti-inflammatory, antiproliferative, and antiangiogenic effects [64]. A meta-analysis comparing dienogest with GnRH analogs found no significant difference in postoperative recurrence rates [65]. However, since irregular vaginal bleeding frequently persists with progestin monotherapy, GnRH analogs may be administered first to suppress bleeding before progestin monotherapy is initiated [66]. Nevertheless, side effects associated with hypoestrogenism may occur even with progestin monotherapy. Although OC has few side effects due to hypoestrogenemia, the risk of thrombosis increases in patients such as the elderly, those with obesity, and smokers [67]. Although dienogest exhibits comparable efficacy to OC, it may have fewer side effects [68]. In clinical practice, the most appropriate hormone therapy is selected based on these characteristics and the patient’s life stage and risk profile.

Although hormone therapy has a strong evidence base, it has several significant limitations. First, it cannot be used in patients desiring pregnancy because it suppresses ovulation. OC administration is restricted in patients with risk factors such as obesity, smoking, and advanced age. Progestins and GnRH analogs can cause side effects, such as hot flashes and mood swings, due to long-term hypoestrogenism. Furthermore, a certain number of patients do not respond to treatment, which may be due to the reduced expression of progesterone receptors and abnormal intracellular signal transduction in diseased tissue. This “progesterone resistance” may lead to persistent symptoms and lesion activity [69, 70]. Furthermore, because hormone therapy is not curative, it has a high rate of relapse of symptoms and lesions when treatment is interrupted due to the desire to conceive.

Given these circumstances, non-hormonal therapies that directly control inflammation, oxidative stress, immune abnormalities, and cell proliferation without altering endogenous hormone secretion are desirable. Currently, various approaches, including immunomodulatory and anti-inflammatory, antifibrotic, and antiangiogenic treatments, are being investigated. For example, inflammatory and oxidative stress control drugs, such as methotrexate, celecoxib, and BAY11-7082 ([1]−3-[4-methylphenyl] sulfonylprop-2-enenitrile), inhibit the NF-κB, COX-2, and ROS pathways, thereby blocking the chronic inflammatory loop [11, 71–73]. mTOR, PI3K, and MAPK inhibitors (rapamycin, LY294002, U0126) inhibit the proliferation and migration of diseased cells and exert antiproliferative effects associated with hormonal activity [8]. Promising antiangiogenic treatments include angiostatin, endostatin, anti-VEGF antibodies, statins, and dopamine agonists [51]. However, these drugs have all only been studied preclinically in vitro or in vivo. Furthermore, methotrexate, celecoxib, anti-VEGF antibodies, and statins pose the risk of teratogenicity and fetal toxicity, contraindicating them for patients desiring pregnancy. Although endometriosis progression is caused by many factors, such as inflammation, oxidative stress, and abnormal intracellular signals, the suitability of single mechanism-based medicines for all cases is unclear. Currently, polyphenols, such as curcumin, epigallocatechin gallate (EGCG), and resveratrol, are among the most promising non-hormonal therapies for endometriosis. In vitro and in vivo studies have demonstrated that these compounds inhibit endometriosis through multiple mechanisms, including anti-inflammatory, antioxidant, antiangiogenic, and anti-fibrotic effects [74]. Clinical trials have also demonstrated that the combined use of curcumin and dienogest reduces endometriosis-associated pain and improves quality of life [75]. However, none of these substances are effective on their own and present low bioavailability [76, 77].

LF may be able to address these clinical challenges. Unlike polyphenols and other drugs, LF is a natural protein that is naturally present in the human body. It is also expressed in the endometrium, cervical mucus, and amniotic fluid, making it possible to administer it to women planning pregnancy or during pregnancy. LF also does not suppress ovulation and has pleiotropic effects that simultaneously regulate inflammation, oxidative stress, and cell proliferation. Although its low bioavailability, similar to that of polyphenols, remains an issue that needs to be addressed, the potential of LF as a new non-hormonal therapeutic candidate for endometriosis warrants further investigation.

### General Characteristics of LF

LF is an iron-binding glycoprotein approximately 80 kDa in length that belongs to the transferrin family and occurs in mammalian exocrine fluids. It was first reported in 1939 as a “red protein” found in bovine milk. In 1960, it was isolated from human and bovine milk, and its amino acid sequence was determined [78]. LF powder is reddish because its two globular domains are linked by a single polypeptide and strongly bind to iron ions [79, 80]. Although some differences in amino acid sequence homology vary across animal species (approximately 70%–80%), recombinant human LF (rhLF) and bovine LF (bLF) have been found comparable in major biological activities such as cell proliferation, antibacterial activity, and immunomodulation [81]. rhLF is derived from the human LF sequence and has low immunogenicity, making it suitable for hypoallergenic applications. In contrast, bLF can be produced in large quantities and at low cost from whey-derived raw materials by filtration and ion exchange purification [82, 83].

Human breast milk has the highest LF concentrations [84]. Furthermore, LF is widely distributed in exocrine fluids such as saliva, tears, and digestive mucus, as well as in endometrial and cervical glands [85].

The main biological actions of LF are classified into the following six categories:

1. Anti-inflammatory: LF is a significant contributor to the innate immunity of mammals [86]. As a major immunoregulatory molecule, LF suppresses neutrophil and macrophage overactivity and reduces the production of cytokines such as IL-1β and TNF-α [87]. It may prevent tissue damage and exacerbation of autoimmune reactions by inhibiting the formation of neutrophil extracellular traps (NETs) [88]. Neutrophils actually accumulate LF in secondary granules and release it rapidly during inflammation or infection [89]. Clinical human studies comparing patients with chronic kidney disease, inflammatory bowel disease, and neonatal necrotizing enterocolitis with healthy adults for immune improvement highlight the anti-inflammatory effects of oral LF administration [19, 90–93]. Furthermore, a preclinical investigation found that LF inhibits PGE2 and COX2 [94, 95].
2. Iron-chelating and antioxidative: The molecular structure of LF has two domains, the N-lobe and C-lobe, each of which binds to Fe^3+^ with high affinity, thereby depriving microorganisms of iron, which is essential for their survival, thereby inhibiting their proliferation. Furthermore, it inhibits ROS production via the Fenton reaction, protecting tissues [96]. The antioxidant properties of LF have been observed in inflamed tissues and confirmed preclinically in various models, including intestinal epithelial cells, hepatocytes, and human umbilical vein endothelial cells, as well as in neonatal ischemic encephalopathy [96–101]. A clinical study on intravaginal LF administration in pregnant women found that LF reduced oxidative stress in amniotic fluid [24].
3. Antiproliferative: LF inhibits cell proliferation through various mechanisms, including suppression of the PI3K/Akt pathways, inhibition of angiogenesis, iron chelation, and antioxidation [26, 102–105]. The antitumor effects of LF have been observed in basic in vitro and in vivo research on cervical [106, 107], endometrial [106], breast [108–112], oral [113–116], and colorectal [25, 117–119] cancers.
4. Antifibrotic: LF has also shown antifibrotic effects in animal models of liver and heart fibrosis, induced through the suppression of the TGF β−1/Smad signaling pathway, inhibition of NF-κB, and reduction of oxidative stress by iron chelation [27, 120–123].
5. Antiangiogenic: In tumor models, LF can inhibit angiogenesis through various mechanisms, including suppression of the VEGF and hypoxia-inducible factor (HIF)−1α expression, and oxidative stress modulation via iron chelation [25, 26, 124–127].
6. Antibacterial: Most bacteria require iron to survive. LF inhibits bacterial growth by reducing free iron levels as well as by directly damaging bacterial cell membranes [128–131]. Clinical trials have demonstrated the efficacy of LF in bacterial vaginosis [132], adjuvant therapy for *Helicobacter pylori* eradication [133–135], periodontal disease management [136], and infection control, including sepsis in low birth weight infants [137–139].

Considering these pleiotropic effects, LF may possibly mitigate endometriosis symptoms and progression.

### LF Concentration in the Body of Patients with Endometriosis

The association between LF and endometriosis has been reported in five human observational studies (Table 1).

**Table 1:** Summary of observational studies investigating lactoferrin levels in patients with endometriosis

Publication year	Study type	Background of the non-endometriosis (control) group	Stage	Menstrual phase	Main findings	PMID
2023	Human serum analysis	Patients with benign gynecological diseases	I: *n* = 15 II: *n* = 4 III: *n* = 18 IV: *n* = 14	Follicular: N/A Luteal: N/A Postmenopausal: 0	Serum LF and anti-LF antibody levels were elevated in patients with stage III/IV endometriosis vs. controls. After surgical resection of endometriotic lesions, anti-LF antibody levels decreased significantly.	36,476,585 [140]
2023	Human plasma and peritoneal fluid analysis	Patients with clinically suspected endometriosis	I: *n* = 18 II: *n* = 7 III: *n* = 23 IV: *n* = 9	Follicular Control: 25 Endometriosis: 35 Luteal Control: 8 Endometriosis: 22 Postmenopausal: 0	Plasma LF was significantly correlated with iron and transferrin levels in patients with endometriosis. Patients with stage IV endometriosis had a significantly lower peritoneal fluid-to-plasma LF ratio in than patients with stage I disease.	36,675,136 [141]
2007	Human peritoneal fluid analysis	Patients with benign gynecological diseases	I: *n* = 16 II: *n* = 18 III or IV: *n* = 15	Follicular: all Postmenopausal: 0	Women with mild endometriosis had lower peritoneal fluid lactoferrin (LF) concentrations than both patients with high classification scores and controls. No significant difference in peritoneal LF levels was found between patients with stage II, III, or IV endometriosis and controls.	16,644,090 [142]
2007	Human peritoneal fluid analysis	Patients with benign gynecological diseases	I: *n* = 10 II: *n* = 0 III: *n* = 5 IV: *n* = 3	Follicular Control: 4 Endometriosis: 10 Luteal Control: 7 Endometriosis: 7 Postmenopausal Control: 2 Endometriosis: 1	LF concentration was significantly lower in patients with stage I endometriosis than in controls and patients with stage III/IV disease.	17,611,835 [143]
2023	Human plasma and peritoneal fluid analysis	Patients with clinically suspected endometriosis	N/A	Follicular: N/A Luteal: N/A Postmenopausal: 0	In endometriotic conditions, LF correlated poorly with vitamin D-binding protein in plasma, whereas they were significantly correlated in peritoneal fluid.	37,175,534 [144]

Endometriosis stage was determined according to the American Society for Reproductive Medicine classification of endometriosis. *LF*, lactoferrin; *N/A*, not available

Serum LF concentrations were similar in patients with early endometriosis compared with those without but were significantly elevated in patients with advanced endometriosis [140]. Skarzynska et al. reported that plasma LF concentrations tended to increase as the stage of endometriosis progressed, although the difference was not significant [141]. When evaluating blood LF concentrations, plasma LF concentrations are generally measured, but as in Mori-Yamanaka et al. [140], serum LF concentrations are used when discussing neutrophil function or neutrophil extracellular trap. In patients with advanced endometriosis, blood LF concentrations may be elevated, reflecting systemic inflammation that is not seen in patients with early endometriosis. Furthermore, the increase in serum anti-LF antibody concentrations in patients with endometriosis and the decrease observed after surgical resection of endometriotic lesions indicate that reducing antigen (lesion) load can reduce autoantibody responses [140]. If the patient’s own antibodies neutralize LF, increasing LF concentrations or decreasing these antibodies may restore LF’s protective function.

We hypothesized that supraphysiological elevation of blood LF concentrations could therapeutically control endometriosis progression. This hypothesis rests on two premises: First, that increased blood LF may act as a host defense response to systemic inflammation; and second, that endogenous antibodies might neutralize LF, impairing its function. Since LF in peritoneal fluid is supplied from the blood, increasing its systemic concentration could enhance its local anti-inflammatory activity, thereby controlling disease progression.

Drawing any consistent conclusions on LF concentrations in peritoneal fluid is currently difficult. A meta-analysis of iron metabolism markers in the peritoneal fluid of patients with endometriosis, including 217 patients from three studies, found no significant differences between the endometriosis and control groups [145]. Individual studies have reported varying results, with Polak et al. [142] and Riley et al. [143] reporting that LF concentrations in peritoneal fluid significantly decreased in certain subgroups of patients with endometriosis compared with patients without endometriosis. In contrast, Skarzynska et al. [141] found no significant difference in LF concentrations in peritoneal fluid between endometriosis and non-endometriosis groups. However, because this study targeted patients clinically suspected of endometriosis, all patients in the non-endometriosis group had infertility, pelvic pain, and ovarian cysts, which is a different comparison from those in other studies. In other studies, since the presence or absence of endometriotic lesions needs to be confirmed through surgery, the non-endometriosis group consisted of patients with benign gynecological diseases, such as ovarian tumors and uterine myomas, and were thus not compared with healthy women. The changes in LF concentration in peritoneal fluid in benign gynecological diseases other than endometriosis are unknown. Furthermore, the backgrounds of the studies are inconsistent, showing differences in the proportions of menstrual cycles and endometriosis stages and inconsistencies in the exclusion of postmenopausal patients. Thus, the results cannot be simply compared, necessitating caution in their interpretation. A report examining vitamin D-binding protein (VDBP) and LF concentrations in plasma and peritoneal fluid found that among patients clinically suspected of endometriosis, plasma VDBP concentrations were correlated with LF concentrations in plasma or peritoneal fluid in patients with specific VDBP levels. No significant difference was observed in plasma or peritoneal LF concentrations between the two groups [144]. However, this study explored diagnostic markers for endometriosis using minimally invasive methods other than surgery, and, like Skarzynska et al., only patients who were clinically suspected of endometriosis were included in the study. In summary, because patient backgrounds varied among studies, no clear conclusions can be drawn regarding the dynamics of LF in peritoneal fluid in endometriosis. However, assuming that the decrease in LF concentration in peritoneal fluid reflects local consumption, as reported in some studies, an increase in LF concentration in peritoneal fluid above physiological levels may suppress endometriosis progression.

### Basic Research on the Effects of LF on Endometriosis

The effect of LF on human endometriotic cells has been demonstrated in only one in vitro study [29]. Bovine LF (1 mg/mL) added to cultured human endometriotic stromal cells significantly suppressed proliferation by inducing cell cycle arrest at the G0/G1 phase. LF treatment also reduced the expression of p-AKT, p-mTOR, and p-S6K, which are major components of the PI3K/Akt/mTOR pathway, and suppressed cell proliferation signals. However, under the same conditions, LF did not suppress the proliferation of eutopic endometrial stromal cells. The promotion of proliferation by LF in human endometrial stromal cells has been previously reported [146] and its selectivity in inhibiting only endometriotic cells confirms its safety and therapeutic efficacy.

Interestingly, although outside the context of direct LF supplementation, a study using a rodent model of endometriosis found an inverse correlation between LF concentrations in peritoneal fluid and endometriotic lesions. Specifically, LF concentrations in peritoneal fluid were significantly higher in exercise-exposed groups than in controls and were inversely correlated with the size of endometriotic lesions. This study also showed that exercise increased ERα expression and decreased ERβ expression in endometriotic lesions and mesenteric fat, indicating that exercise may improve the abnormalities in estrogen receptor expression that occur in endometriosis [147].

In response to the decreased LF concentration in peritoneal fluid and increased serum anti-LF antibody observed in patients with endometriosis, supplementation with supraphysiological concentrations of LF may help suppress the progression of endometriosis.

### Potential but Unverified Non-Hormonal Therapeutic Effects of LF in Endometriosis

**Fig. 2:**
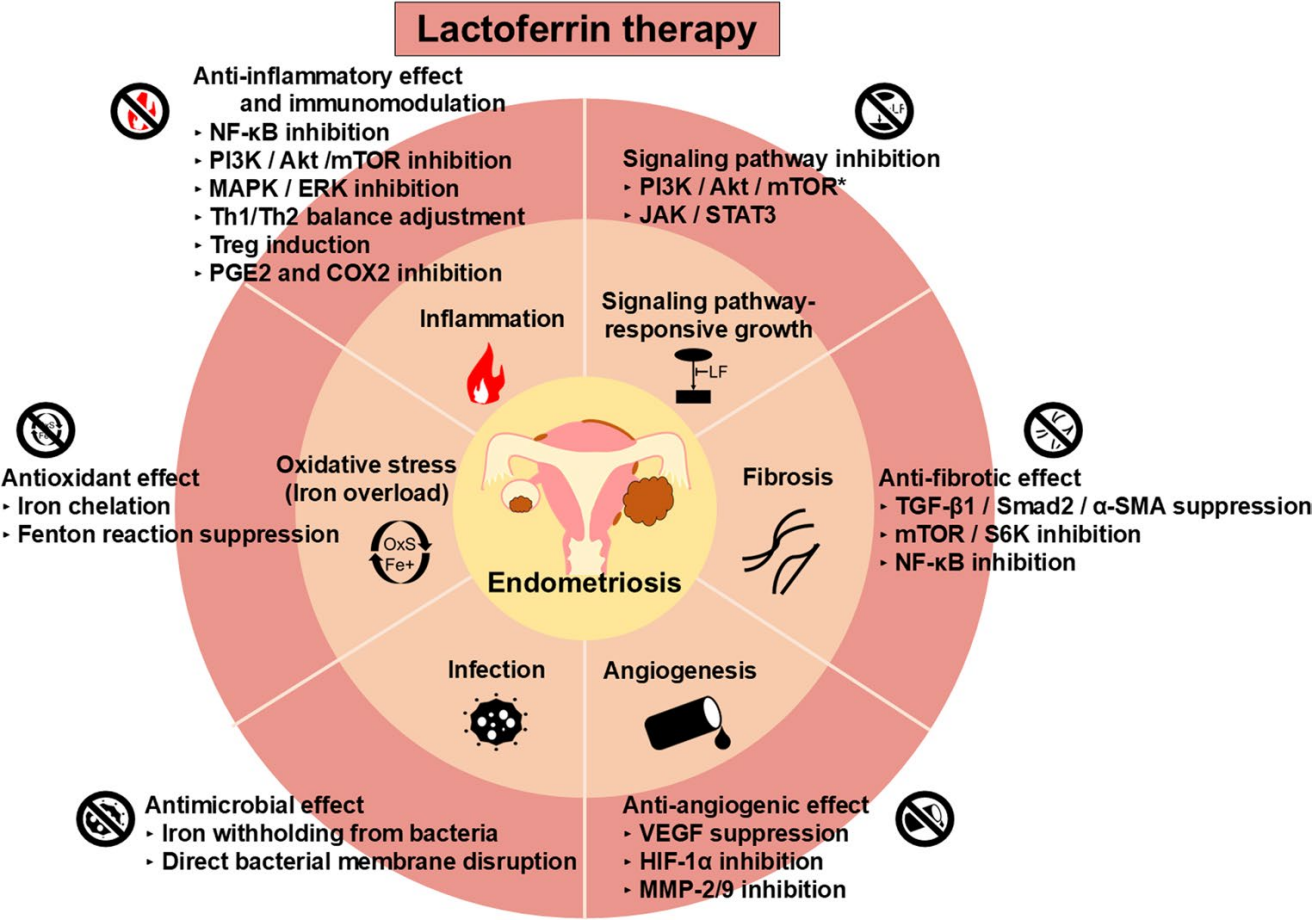
Potential therapeutic mechanisms of lactoferrin (LF) in endometriosis The figure illustrates the potential therapeutic effects of LF for endometriosis. Although these effects are based on the general characteristics of LF and factors that may be involved in the pathogenesis and progression of endometriosis, the direct effect of LF on endometriosis requires further investigation *Effects demonstrated in preclinical studies on endometriosis. *NF-κB*, nuclear factor-kappa B; *PI3K*, phosphatidylinositol 3-kinase; *Akt*, protein kinase B; *mTOR*, mammalian target of rapamycin; *MAPK*, mitogen-activated protein kinase; *ERK*, extracellular signal-regulated kinase; *Th1*, T helper 1 cell; *Th2*, T helper 2 cell; *Treg*, regulatory T cell; *PGE2*, prostaglandin E2; *COX2*, cyclooxygenase-2; *VEGF*, vascular endothelial growth factor; *HIF-1α*, hypoxia-inducible factor 1α; *MMP*, matrix metalloproteinase; *TGF-β1/Smad2/α-SMA*, transforming growth factor beta 1/SMA and MAD homologs/α-smooth muscle actin; *S6K*, ribosomal protein S6 kinase; *JAK/STAT3*, Janus kinase/signal transducer and activator of transcription 3

Here, we list the potential mechanisms of action of LF that may be involved in the progression of endometriosis (Fig. 2) but for which direct evidence is still lacking.

No studies have demonstrated the anti-inflammatory or immunomodulatory effects of LF in endometriosis. However, it has exhibited anti-inflammatory effects in chronic endometritis, which is characterized by chronic inflammation of eutopic endometrium, expecting similar effects in endometriosis. In an in vitro experiment using cultured human endometrial stromal cells (ESCs) with chronic endometritis, the addition of bovine LF (1 mg/mL) significantly decreased TNF-α and IL-1β expression [148]. Furthermore, in vitro and in vivo experiments have demonstrated that LF indirectly suppresses IL-6 and IL-1β production by suppressing phosphorylation in the AKT and MAPK (ERK) pathways and reducing NF-κB activation [149–151]. LF also suppresses neutrophil and macrophage overactivation, as well as excessive cytokine release and the formation of NETs, thereby also possibly preventing the enhancement of local inflammation [88, 152]. In endometriosis, the Th1-dominant immune balance and abnormal regulatory T cell (Treg) function promote the formation and proliferation of diseased lesions [153–155]. LF may restore immune homeostasis by inducing differentiation of naive T cells into Treg and adjusting the Th1/Th2 balance, suppressing lesion formation and instead promoting their removal [156–160]. Furthermore, decreased intestinal flora diversity and increased lipopolysaccharide (LPS) load promote systemic inflammation in endometriosis [31, 161]. LF may suppress systemic inflammation in endometriosis by restoring vaginal [16, 22] and intestinal flora [162–164]. Furthermore, increased COX2 and PGE2 expression in endometriosis has been reported [41–43]. LF exhibits analgesic activity by interacting with the low-density lipoprotein receptor-related protein, suppressing COX2 expression, and inhibiting PGE2 production in human chondrocytes [94, 95]. LF may suppress COX2 and PGE2 production while also exerting analgesic effects in endometriosis. Considering the risk of fetal toxicity of non-steroidal anti-inflammatory drugs, which are direct COX2 and PGE2 inhibitors, partial COX2 and PGE2 inhibition by LF merits research attention. Future studies should explore the anti-inflammatory effects of LF and elucidate its effects on endometriosis.

The iron-chelating effect of LF in endometriosis has not yet been reported. However, human clinical studies have shown that advanced endometriosis is characterized by high iron and ferritin levels in peritoneal fluid (reflecting iron overload) but not in plasma. Furthermore, the peritoneal fluid-to-plasma LF ratio is lower in r-ASRM (Revised-American Society for Reproductive Medicine) stage IV endometriosis than in stage I, indicating that the innate protective effect of LF is relatively reduced in advanced endometriosis [141]. Furthermore, desferrioxamine (DFO), another iron chelator, has shown efficacy in endometriosis models, suggesting a similar effect by LF. DFO is a low-molecular-weight (approximately 656 Da) hexadentate iron chelator with an extremely high binding constant for Fe³⁺, potently and selectively sequestering free iron. DFO is used clinically via intraperitoneal or intravenous administration, rapidly reducing free iron to treat iron overload disorders, such as thalassemia [165]. DFO chemically sequesters free iron, whereas LF stabilizes iron as a protein complex. However, pharmacologically, LF, like DFO, may exhibit antioxidant and antiproliferative effects via its iron-chelating activity [96]. Indeed, in mouse endometriosis models, DFO administration reduced the iron content and proliferative activity of diseased tissue, reversing proliferation induced by high iron dosing [166–168]. Furthermore, follicular fluid from patients with endometriosis contains excess iron [168]. The addition of DFO to embryo culture medium improved embryo development in mice [169]. These findings suggest that free iron sequestration by LF may improve embryo quality and reproductive outcomes. Therefore, future studies should elucidate the iron-chelating activity of LF and its impact on endometriosis.

In endometriosis, tissue fibrosis occurs through TGF-β1/Smad signaling-dependent myofibroblast activation [52]. LF suppresses inflammatory responses via the mTOR/S6K pathway and has exhibited antifibrotic effects in heart fibrosis models [27]. LF ameliorates hepatic fibrosis in rats by attenuating the TGF-β1/Smad2/α-SMA and NF-κB signaling pathways [120–123]. Thus, empirical verification of the antifibrotic effects of LF in endometriosis would be desirable.

The antiangiogenic effect of LF is also a promising therapeutic mechanism in endometriosis; however, direct verification has not yet been conducted. LF suppressed angiogenesis in malignant tumor models by downregulating VEGF and HIF-1α expression and by regulating antioxidant stress through iron chelation [25, 26, 124–127]. LF can indirectly inhibit angiogenesis by suppressing matrix degradation through the reduction of MMP-2/−9 activity [170]. Furthermore, LF may affect various angiogenic pathways, including FGF signaling, through its antioxidant activity [96]. These findings signify the ability of LF to regulate ectopic tissue invasion and angiogenesis in endometriotic lesions.

Some bacteria have been associated with the pathogenesis and progression of endometriosis, with infection possibly inducing inflammation and adhesions [55, 56]. The relationship between endometriosis and bacterial infections has been reported in *Fusobacterium*, *Mycoplasma genitalium*, and *Ureaplasma urealyticum* [171–173]. Although the antibacterial effect of LF on endometriotic lesions has not been established, it has been demonstrated in other female reproductive tract diseases and inflammatory models [132, 174, 175]. Theoretically, local LF concentrations at the lesions could reach sufficient levels for inhibiting bacterial growth, which would be a worthwhile direction for future investigations.

Clinical studies have shown that LF concentrations in peritoneal fluid from patients with endometriosis are highly correlated with plasma concentrations of VDBP, a type of inflammatory protein, which has otherwise no correlation with plasma LF concentrations [144]. Therefore, the use of LF as a biomarker may require assessments of VDBP and other immune-related factors.

### Association of LF with the Endocrine System

This section explores the interactions of LF with the endocrine system, particularly with estrogen, and discusses the potential implications of administering LF in combination with or as an alternative to hormone therapy for endometriosis. Estrogen directly promotes LF gene expression, and serum estrogen levels are positively correlated with LF expression in the cervical mucus, endometrium, and vaginal epithelium in multiple species, including humans, mice, monkeys, and dogs [176–181]. Moreover, reduced estrogen decreases LF production in these tissues and weakens the local innate immune barrier, which is associated with an increased risk of infection and inflammatory diseases [181, 182].

These findings demonstrate that in treating endometriosis, a low-estrogen environment induced by GnRH analogs or progestins may instead promote disease progression by reducing endogenous LF and suppressing immune defense and anti-inflammatory effects. Therefore, exogenous LF supplementation may help maintain and complement LF function under hormone therapy and boost therapeutic effects.

However, no studies have demonstrated the efficacy of combining hormone therapy and LF supplementation for human endometriosis, necessitating further basic research for verification.

### Other Benefits of Using LF for Endometriosis

Although the evidence level for the benefits of LF treatment in endometriosis remains low, several human clinical trials have demonstrated the effectiveness of LF treatment in the female reproductive tract, assisted reproductive technology, and perinatal care, indicating various positive secondary benefits of LF therapy for endometriosis.

Some clinical studies have shown that LF improves perinatal outcomes, including a significant reduction in the risk of preterm birth [18, 21, 174]. A pilot randomized control trial showed that vaginal administration of LF significantly reduced oxidative stress markers and inflammatory cytokines in amniotic fluid, suggesting a risk reduction of inflammatory complications [24].

In endometriosis, the endometrial microbiome may be altered [183]. LF supplementation improved reproductive outcomes in women with a history of repeated implantation failure and a non-*Lactobacillus*-dominated bacterial flora in vaginal secretions and the endometrium [16]. In this study, it is interesting to note that oral administration of LF improved the bacterial flora in vaginal secretions and endometrium. LF is also produced by theca cells and can influence follicle maturation. A report demonstrated that high LF concentrations in follicular fluid improved reproductive outcomes, whereas another found no effects on reproductive outcomes [184, 185].

Intervention studies have confirmed the efficacy of oral LF administration for anemia in pregnant and non-pregnant women through its ability to restore ferroportin-mediated iron export from cells to the blood [186]. Furthermore, two clinical trials have demonstrated the efficacy of LF for bacterial vaginosis in non-pregnant women [22, 132].

Evidence of the anti-inflammatory effects of LF in endometritis is limited to preclinical studies. In vitro, LF suppressed the expression of TNF-α and IL-1β in ESCs isolated from patients with human chronic endometritis [148]. In equine endometritis, LF intrauterine injection after mating reduced polymorphonuclear leukocyte infiltration and increased levels of the anti-inflammatory cytokine IL-1RN [187]. Furthermore, in a mouse model of LPS-induced acute endometritis, intraperitoneal LF administration significantly suppressed histological damage, myeloperoxidase activity, nitrous oxide production, and activation of the NF-κB signaling pathway [20].

Although some disagreement exists regarding ovarian dysfunction caused by endometriosis itself [188], most studies confirm it [189, 190]; thus, protecting ovarian function in endometriosis is an important issue. Although no reports have investigated the therapeutic effect of LF in ovarian dysfunction associated with endometriosis, a series of animal model studies have provided valuable insights. Specifically, in endometriosis, activation of the PI3K/Akt/mTOR pathway causes excessive development of primordial follicles and impaired ovarian function [191]. Similarly, partial oophorectomy promotes follicular development via the mTOR pathway [192]. However, LF inhibits the PI3K-PTEN-Akt-mTOR pathway in endometriosis (basic research) [29], kidney disease [193], neurological diseases [194], and certain cancers (basic and clinical research) [115, 119]. Furthermore, ovarian dysfunction caused by cyclophosphamide, an anticancer drug with high ovarian toxicity, entails excessive follicle loss induced through activation of the PI3K-PTEN-Akt-mTOR pathway [195, 196]. In contrast, the combination of cyclophosphamide and LF protects ovarian function [197, 198]. Although direct evidence is still lacking, the findings of the above studies indicate that the decline in ovarian function caused by activation of the PI3K/Akt/mTOR pathway in endometriosis may be reversed by LF.

#### Endometriosis Treatment Strategies by Life Stage: Conventional Treatment and Future Positioning of LF

**Fig. 3:**
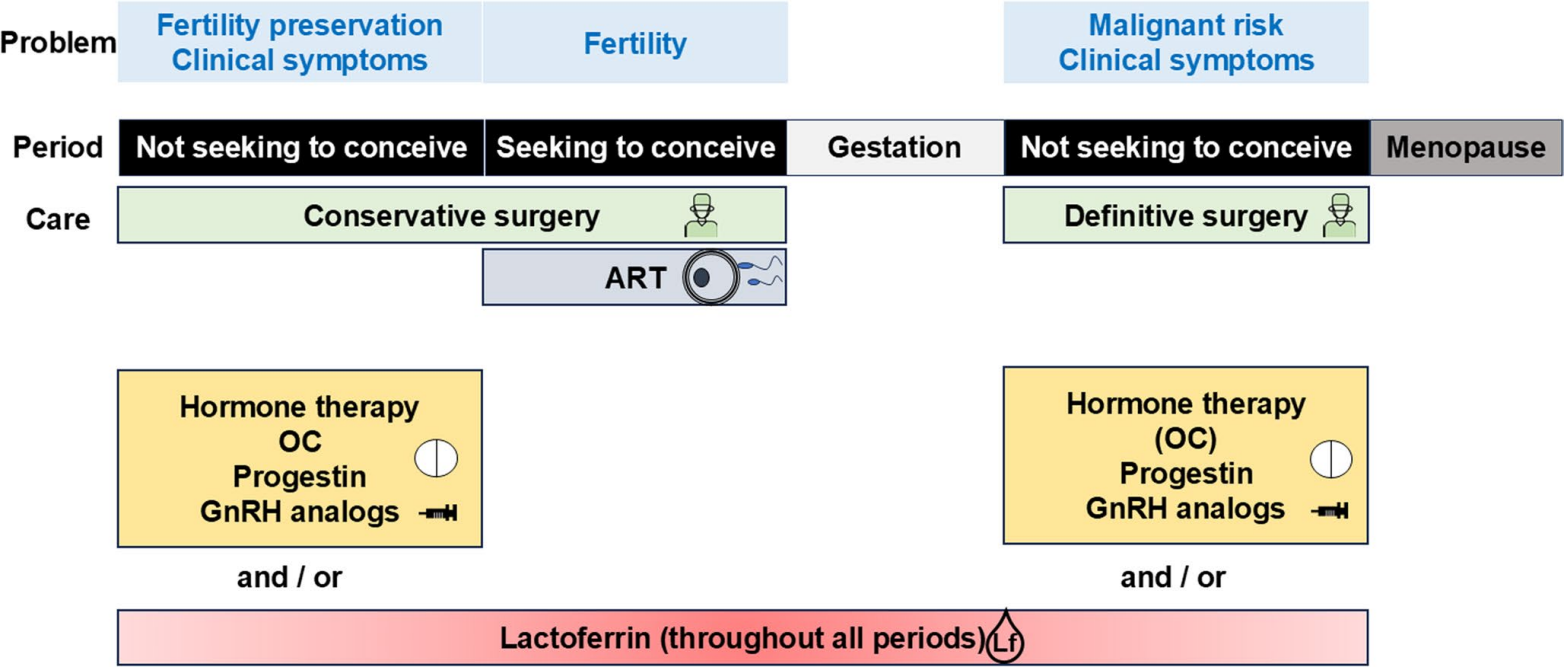
Proposed treatment strategy for endometriosis by lactoferrin (LF) at different life stages including gestation Problems associated with endometriosis and treatments at each life stage. For women with no plans for future conception, radical surgery is the most effective. However, for those who wish to preserve their fertility, surgical treatment is a low priority due to the risk of decreased ovarian function and recurrence. Hormone therapy (oral contraceptives [OC], progestin, and gonadotropin-releasing hormone [GnRH] analogs) suppresses ovulation, restricting continued use in patients desiring pregnancy. LF does not suppress ovulation and, as a non-hormonal alternative that can be safely taken by women who wish to conceive or are pregnant, can potentially provide seamless endometriosis treatment at all life stages, including gestation. *ART*, assisted reproductive technology

Although evidence for LF administration for endometriosis remains limited to preclinical studies, clinical trials have demonstrated improved reproductive and perinatal outcomes (Table 2) [16, 18, 22, 24, 132, 175, 187, 199–203]. LF has an extremely low risk of teratogenicity and fetal toxicity, making it suitable for administration at all life stages (Fig. 3). In particular, for cases in which hormone therapy is contraindicated or resistant to hormone therapy, LF may be a promising option as a non-hormonal alternative or adjunctive therapy. Furthermore, LF may offer a safe and seamless treatment option for pregnant women and those planning pregnancy, a period currently lacking satisfactory treatment options in the pharmacotherapy of endometriosis.

**Table 2:** Clinical studies demonstrating the effectiveness of lactoferrin on female reproductive health and pregnancy outcomes

Publication year	Study Type	Target Outcome	Summary of Findings	PMID
2023	Systematic review & meta-analysis	Perinatal outcomes (preterm birth prevention)	LF supplementation reduced the risk of preterm birth < 37 weeks (OR: 0.43; 95% CI: 0.2–0.9) and increased gestational age at delivery (mean difference 0.46 weeks, SD = 0.17, *P* = 0.006; 6 trials, 333 women).	35,333,034[18]
2021	Retrospective cohort study	Perinatal outcomes (preterm birth prevention)	300 mg/day vaginal LF administration lowered preterm birth rate and prolonged gestation compared with controls.	31,722,591[175]
2020	Open-label pilot RCT	Perinatal outcomes (amniotic fluid oxidative stress)	Single 300 mg vaginal dose reduced oxidative stress markers and proinflammatory cytokines in amniotic fluid.	33,015,104[24]
2017	Open-label RCT	Perinatal outcomes (amniotic fluid cytokine)	Single 300 mg vaginal LF downregulated 17 proinflammatory amniotic mediators while upregulating 7 anti-inflammatory amniotic mediators.	28,289,333[200]
2016	RCT (Obstetricians and research assistants were blinded)	Perinatal outcomes (amniotic fluid cytokine and MMP)	PGE2, active MMP-9, and its inhibitor TIMP-1 were lower in LF-treated (single 300 mg vaginal administration) group than in controls. Conversely, active MMP-2 and MMP-2/TIMP-2 molar ratio were increased.	27,872,513[201]
2014	Open-label RCT	Perinatal outcomes (amniotic fluid cytokine)	Single 300 mg vaginal LF decreased amniotic interleukin-6 concentration.	24,642,648[202]
2022	Prospective cohort study with pilot interventional supplementation trial	Reproductive outcomes in women with dysbiosis and repeated implantation failure	Oral enteric‑coated LF (700 mg/day for ≥ 28 days) normalized genital microbiota in 43.2% of patients with RIF, who then achieved higher live birth rates in vitrified‑warmed embryo transfer cycles (57.1% vs. 11.1%; OR: 10.7; *P* = 0.046).	35,987,819[16]
2018	Interventional before–after study	Hematologic markers in pregnant women with anemia	Oral bovine LF (100 mg twice daily) administered until delivery effectively treated pathological anemia during pregnancy.	30,298,070[187]
2017	Systematic review & meta-analysis	Treatment of iron deficiency anemia	Increased hemoglobin levels at 4 weeks after daily oral LF compared with daily oral ferrous sulfate (mean difference 0.77; 95% CI 0.04–1.55; *P* = 0.04; 4 trials, 600 women). Significantly less gastrointestinal side effects reported with LF treatment.	29,059,584[203]
2017	Open-label RCT	Bacterial vaginosis resolution & microbiota balance	Both 100 mg and 200 mg vaginal bovine LF pessaries × 10 days significantly reduced vaginosis-associated bacteria (*Gardnerella*, *Prevotella*, etc.) and increased *Lactobacillus* spp.; The 200 mg LF dose maintained *Lactobacillus* dominance for ≥ 2 weeks post-treatment	28,959,181[22]
2019	Double-blind, placebo-controlled RCT	Bacterial vaginosis symptom relief & recurrence prevention	Metronidazole + LF-containing probiotic tablet (10 days) vs. metronidazole + placebo: the LF-probiotic arm showed higher Nugent score normalization, greater symptom resolution, and significantly lower 6-month recurrence rates	30,525,953[132]
2025	Open-label RCT	Prevention of recurrent urinary tract infections	Oral LF (100 mg twice daily) until delivery significantly reduced the incidence of asymptomatic bacteriuria and acute cystitis in the LF-treated group compared with the control group.	40,264,102[204]

*RCT* randomized controlled trial, *LF* lactoferrin, *CI* confidence interval, *SD* standard deviation, *MMP* matrix metalloproteinase, *RIF* repeated implantation failure, *TIMP* tissue inhibitor of metalloproteinase

### Issues and Future Research Directions for LF Treatment for Endometriosis

To realize the clinical application of LF for endometriosis, it is first necessary to clarify the direct mechanisms of action of LF in endometriosis, such as its effects on iron metabolism, oxidative stress, inflammation, and angiogenesis, through preclinical studies. Specifically, it is necessary to compare the therapeutic effects in vitro and in vivo among the control, hormone therapy alone, LF alone, and hormone therapy and LF group, and to consider in what situations the clinical application of LF is likely to be feasible. Subsequent pilot-scale proof-of-concept studies aimed at clinical application are desired in the setting of LF alone or in combination with hormone therapy.

One of the challenges currently anticipated for clinical application of LF is its bioavailability. Unfortunately, the bioavailability of simple oral administration of LF is estimated to be low, and some kind of innovation is required. For example, oral administration of 3 g bovine LF per day to patients with colonic polyps increased serum human LF concentrations by only 20 ng/mL [204]. Orally administered LF is largely degraded during passage through the stomach and small intestine in adults, resulting in a bioavailability of less than 1% [205]. LF is mostly resistant to degradation in newborns and infants whose digestive enzymes are still immature [206]. However, when considering administration to adults, enteric coating is necessary to preserve it until it reaches the small intestine where LF receptors are present. This technology has already been established, and most LF supplements currently on the market feature this coating. However, even if LF can be absorbed from the gastrointestinal tract and enter systemic circulation, the half-life of free LF in plasma is short, ranging from 12 to 60 min, requiring further ingenuity in its delivery [207]. To enhance the bioavailability and prolong the half-life of LF, active research is being conducted into improving local LF delivery using DDS technologies, such as liposomes, solid lipid nanoparticles, nanostructured lipid carriers, polymeric nanoparticles, and hydroxyapatite nanocrystals [207–210]. If these technologies can be used to deliver sufficient concentrations of orally administered LF to endometriotic lesions in vivo, this could facilitate the conduct of clinical trials on oral LF administration, which is the least invasive and most acceptable. To do this, LF must be administered orally or intraperitoneally to endometriosis model mice, and the LF concentration in the blood and in the diseased tissues must be measured. Even if oral LF administration results in only a small change in blood concentration, LF may exert its therapeutic effects through its interactions with lymphatic tissue in the gastrointestinal tract or through the accumulation of absorbed LF in target tissues [207]. The pharmacokinetics of absorbed LF must be clarified using fluorescently labeled LF. The LF concentration that can inhibit the proliferation of endometriotic stromal cells in vitro is 1 mg/mL [29]. The investigation of the other effects of LF on endometriosis needs to begin with in vitro validation.

Another challenge in the clinical application of LF is determining the optimal administration route. If oral LF administration is unable to deliver sufficient LF concentrations to endometriotic lesions in vivo, even with the use of DDS, alternative administration routes will need to be considered. In rodent studies, LF has been administered intraperitoneally and intravenously as an alternative to oral administration [211]. LF is a water-soluble protein that is easily soluble up to approximately 10 mg/mL. The highest LF concentration in humans is approximately 5 mg/mL in colostrum [84]. This indicates that high LF concentrations are unlikely to have adverse effects on normal tissues in vivo, indicating the feasibility of local LF administration. Specifically, LF is effective against endometritis, cervicitis, and vaginitis. Since these diseases are often associated with endometriosis, the efficacy of treatment via transvaginal and intrauterine administration should be investigated in these diseases, which are also often associated with endometriosis [199]. Although it is a more invasive method, local administration of LF following transvaginal ultrasound-guided aspiration of ovarian chocolate cysts may be considered, as it can deliver high concentrations of LF directly to endometriotic lesions.

## Conclusion

LF exerts pleiotropic effects that can simultaneously regulate multiple pathways involved in the pathology of endometriosis, such as inflammation, oxidative stress, abnormal cell proliferation, and infection risk, without suppressing ovulation. It has an extremely low risk of teratogenicity or fetal toxicity and may even improve reproductive and perinatal outcomes. LF may present a promising new non-hormonal therapy as an alternative or adjunct to hormone therapy. Evidence is currently limited on the therapeutic efficacy of LF, even in basic research, which is needed to establish the clinical feasibility of LF therapy. However, LF therapy holds great research value. Future studies should explore its therapeutic mechanisms through in vitro and in vivo studies and determine optimal administration routes and dosage. Furthermore, its safety and efficacy will need to be verified through pilot clinical trials.

## Data Availability

Data generated or analyzed during this study are available from the corresponding author upon reasonable request.
